# Lateral Intercostal Artery Perforator (LICAP) Flap for Level II Oncoplastic Breast Reconstruction: Our Initial Experience

**DOI:** 10.3390/jpm15100491

**Published:** 2025-10-14

**Authors:** Gianluca Marcaccini, Claudia Biagini, Benedetta Daicampi, Simone Miccoli, Pietro Susini, Ishith Seth, Warren M. Rozen, Roberto Cuomo, Luca Grimaldi, Leonardo Barellini

**Affiliations:** 1 Operative Unit of Oncological and Reconstructive Surgery of the Breast, Livorno Hospital, 57100 Livorno, Italy; c.biagini@student.unisi.it (C.B.); benedetta.daicampi@uslnordovest.toscana.it (B.D.); simone.miccoli@uslnordovest.toscana.it (S.M.); leonardo.barellini@uslnordovest.toscana.it (L.B.); 2 Department of Plastic and Reconstructive Surgery, University of Siena, 53100 Siena, Italy; susinipietro@gmail.com (P.S.); robertocuomo@outlook.com (R.C.); luca.grimaldi@unisi.it (L.G.); 3 Faculty of Medicine and Surgery, Central Clinical School, Monash University, Melbourne, VIC 3004, Australia; 4 Department of Plastic and Reconstructive Surgery, Peninsula Health, Frankston, VIC 3199, Australia

**Keywords:** LICAP flap, chest wall perforator flap, personalized oncoplastic breast surgery, individualized volume replacement, breast-conserving surgery, tailored surgical approach

## Abstract

**Background:** Breast-conserving surgery (BCS) combined with radiotherapy achieves oncologic outcomes comparable to mastectomy while preserving breast integrity. However, resections of more than 20% of breast volume or those in challenging quadrants may compromise cosmetic results. Level II oncoplastic techniques using volume replacement flaps aim to address this. The lateral intercostal artery perforator (LICAP) flap is a reliable, muscle-sparing option for lateral and central–lateral breast defects. This study reports our initial experience with LICAP in Level II oncoplastic breast reconstruction. **Methods:** A retrospective review was conducted of women undergoing BCS with LICAP reconstruction between March 2024 and March 2025. The primary outcome was flap-related complications within 90 days. Secondary outcomes included operative time, hospital stay, donor-site morbidity, and six-month aesthetic results using the Harvard scale and BREAST-Q^®^ module. **Results:** Nine women underwent LICAP reconstruction. All tumours were ≤pT2 with negative margins. Mean operative time was 128 min, and the median hospital stay was 2 days. One minor flap-related complication (seroma, 11%) occurred, which was managed conservatively without re-operation or delay in adjuvant therapy. At six months, all patients achieved good or excellent Harvard scores. The mean BREAST-Q^®^ satisfaction score was 79 ± 12. **Conclusions:** LICAP reconstruction is safe, efficient, and provides reliable early aesthetic and patient-reported outcomes. Its low complication rate, high satisfaction, and minimal morbidity support its broader adoption, while larger prospective studies are needed to assess long-term results and refine indications. These findings also underline the role of LICAP reconstruction as part of a personalized surgical strategy, where the choice of technique is tailored to individual anatomy and expectations.

## 1. Introduction

Breast-conserving surgery (BCS), when combined with adjuvant radiotherapy, provides equivalent oncologic outcomes to mastectomy for early-stage breast cancer, with the added benefit of preserving breast integrity and patient self-image [1]. However, excision of greater than 20% of breast volume or tumours located in challenging quadrants can result in poor cosmetic outcomes, including contour deformities, nipple–areolar complex displacement, and volume asymmetry, particularly following radiotherapy [2,3]. Up to one-third of women report dissatisfaction with breast appearance after BCS, with body image concerns continuing to impact long-term psychosocial well-being [4].

Oncoplastic breast surgery (OBS) evolved to address this aesthetic compromise, integrating oncologic resection with immediate breast reshaping or reconstruction. Clough’s widely adopted classification system divides OBS into Level I procedures (mobilisation of local tissue for minor defects) and Level II techniques, which address larger resections through either volume displacement or volume replacement strategies [2]. Volume displacement techniques are effective in large, ptotic breasts but may necessitate contralateral surgery to achieve symmetry and are often unsuitable for smaller-breasted patients. Volume replacement with autologous tissue offers a logical and aesthetic alternative for these patients. In line with the principles of precision medicine, oncoplastic techniques should not be considered universal solutions but rather tools to be tailored to individual patient characteristics. The choice of flap type, skin design, and extent of resection must take into account breast morphology, tumor location, oncologic requirements, and aesthetic expectations. This individualized approach has become a cornerstone of modern reconstructive breast surgery, where success is no longer defined solely by oncologic safety but also by the balance between functional outcomes and cosmetic harmony.

Chest-wall perforator flaps, particularly the lateral intercostal artery perforator (LICAP) flap, have become increasingly favoured in Level II OBS due to their ability to replace lateral and central breast volume without sacrificing muscle [5]. The LICAP flap is based on consistent perforators that arise from the posterior intercostal arteries, typically between the fifth and eighth intercostal spaces, and can be harvested with the patient in a supine position, thereby improving operative efficiency and reducing intraoperative repositioning [6]. Donor site morbidity is minimal, with the scar typically concealed along the bra line and shoulder function fully preserved.

Clinical outcomes with LICAP flaps are promising. Studies report low complication rates (<10%), short operative times (~40–60 min), and high patient satisfaction scores, with favourable outcomes maintained even in patients undergoing adjuvant radiotherapy [7,8]. Comparative analyses have suggested that LICAP flaps are technically easier and more reproducible than alternatives such as the thoracodorsal artery perforator (TDAP) flap, with fewer requirements for preoperative Doppler marking and reduced operative complexity [9].

Despite these encouraging results, most published data on LICAP flaps are limited to small, retrospective single-institution series with short follow-up durations. Modifications to the technique have recently emerged to improve aesthetic results and address defects in less favourable tumour locations, but standardization remains limited, and high-level evidence is lacking. From an oncologic perspective, achieving clear surgical margins remains the paramount objective in breast-conserving surgery. The ability of perforator flaps to restore breast contour without compromising the extent of resection reinforces their role in enabling wider excisions while maintaining cosmesis. This dual advantage strengthens their position within the spectrum of breast-conserving strategies and underscores the value of integrating reconstructive planning into oncologic decision-making.

This study presents our initial experience using the LICAP flap for Level II volume replacement in oncoplastic breast reconstruction. We describe patient selection, surgical planning, flap design, and perioperative management. Additionally, we report early complication rates, time to adjuvant treatment, and aesthetic outcomes at six months. By contributing to the growing body of evidence, we aim to support wider adoption of LICAP flaps and inform best practices for safe, effective, and cosmetically sound breast conservation. This perspective fits within the broader concept of personalized medicine, where surgical planning is adapted to each patient’s oncologic profile, breast morphology, and reconstructive needs.

## 2. Materials and Methods

### 2.1. Study Design and Ethical Considerations

A retrospective review was conducted of all patients who underwent oncoplastic breast-conserving surgery with a lateral intercostal artery perforator (LICAP) flap between 1 March 2024 and 31 March 2025 at our institution: the operative unit of oncological and reconstructive surgery of the breast, of Livorno Hospital. According to national regulations and the hospital’s research policy, retrospective studies of standard procedures that collect only fully anonymised data are exempt from ethics committee approval. The study was therefore performed in accordance with the Declaration of Helsinki without additional Institutional Review Board clearance.

### 2.2. Patient Selection

**Figure 1:**
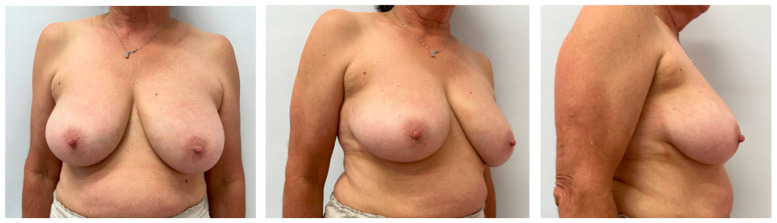
Preoperative photos. Example of a patient candidate for breast reconstruction using a LICAP flap following right superouterine quadrantectomy. The patient has a medium-sized breast, a slight ptosis, and sufficient thoracic fatty tissue.

Eligible patients were women aged ≥18 years with unifocal invasive ≤cT2 carcinoma or ductal carcinoma in situ, with the tumor located in the upper-outer quadrant or the central–lateral breast, and no history of prior breast or chest-wall irradiation [Figure 1]. Exclusion criteria included multicentric disease, indication for mastectomy, or synchronous contralateral cancer. Although neoadjuvant chemotherapy (NACT) was generally an exclusion criterion, one patient who underwent NACT and met all other inclusion criteria was included in the analysis. In total, nine consecutive patients were enrolled.

### 2.3. Surgical Technique

Preoperative markings were performed with the patient in the standing position, outlining the lateral inframammary fold. In the operating room, with the patient in the supine position, local perforators were identified using a colour-Doppler ultrasound scan (Philips Affinity 70 G) equipped with a linear transducer (L 18-5) and a pulse repetition frequency (PRF) set at low values (4 to 8 KHz). This evaluation allowed the surgeon to accurately map the perforator vessel in terms of length, course, and spatial relationship to the planned flap. Additionally, a standard ultrasound scan was used to localize the index lesion, enabling a focused surgical excision with adequate margins [10].

Under general anaesthesia, a skin incision was made along the lateral inframammary fold, and the breast parenchyma was dissected from the subcutaneous tissue to expose and excise the tumour. Subsequently, one to three dominant perforators from the fifth to the eighth intercostal space were skeletonised over 2 to 3 cm while preserving the underlying muscle. The flap, consisting of skin, subcutaneous fat, and fascia, was elevated in a suprafascial plane towards the anterior axillary line. Before flap rotation, a dermal bleeding assessment was performed by making small incisions on the flap surface to confirm the presence of bright red, arterial bleeding. After de-epithelialization, dermal perfusion was further evaluated to ensure adequate vascular supply. Once viability was confirmed, the flap was rotated on its perforator pedicle and tunnelled into the partial mastectomy defect.

After meticulous haemostasis, the donor site was closed primarily without tension. A single 15 Fr closed-suction drain was placed in both the donor and recipient sites and removed when output fell below 30 mL over 24 h. All procedures were performed according to previously described oncoplastic surgical principles. A step-by-step video of the surgical technique is provided in Supplementary Video S1.

### 2.4. Outcome Measures

Primary outcome: flap-related complications within 90 days, categorised as partial necrosis, total loss, haematoma requiring evacuation, infection, or seroma.

Secondary outcomes: operative time, postoperative length of stay, need for revision surgery, donor-site morbidity, and aesthetic result at 6 months assessed by the Harvard four-point scale (excellent/good/fair/poor) plus patient satisfaction using the BREAST-Q^®^ Version 2.0 Breast-Conserving Therapy Module (0–100). Demographic, oncologic, and perioperative variables were extracted from electronic medical records. Follow-up examinations were scheduled at postoperative days 7 ± 2, 30 ± 5, 90 ± 7, and 180 ± 14.

### 2.5. Statistical Analysis

Given the small sample size (n = 9), only descriptive statistics were calculated manually. Continuous variables are presented as mean ± standard deviation or median (interquartile range), and categorical variables as counts and percentages. No hypothesis testing was performed.

## 3. Results

Between 1 March 2024 and 31 March 2025, nine consecutive women underwent breast-conserving surgery with a LICAP flap. Median age at surgery was 55 years (range 37–68), and median body mass index (BMI) was 24.3 kg/m^2^ (interquartile range [IQR] 22.1–27.6). Three of nine patients (33%) were current smokers, and two (22%) had insulin-treated type 2 diabetes. Tumours were located in the upper–outer quadrant in two breasts and in the central–lateral quadrant in seven, with a median pathological diameter of 17 mm (IQR 12–22). All lesions were invasive carcinoma not exceeding pT2 stage. Surgical margins were microscopically negative in every case, with a median clearance of 4.5 mm (IQR 3–6). Sentinel-node biopsy recovered a median of two nodes (range 1–3), with no metastases detected.

The mean operative time was 128 ± 15 min, and the estimated blood loss was 85 ± 20 mL. All procedures were completed under general anaesthesia, without intraoperative complications or conversion to alternative flaps. Perforator dissection revealed a median of two sizeable vessels (range 1–3), originating from the 5th to the 8th intercostal spaces. Drains were removed after a median of 3 days (range 2–4), once output had fallen below 30 mL per 24 h. Patients were discharged after a median of 1 postoperative night. Baseline characteristics, intraoperative metrics, and early outcomes are summarized in Table 1.

During the 90-day postoperative period, one flap-related complication was recorded. There were no cases of partial necrosis, total flap loss, haematoma, surgical-site infection, or wound dehiscence. None of the patients required re-operation, readmission, or antibiotic therapy. A single postoperative seroma was recorded in a patient who had undergone right upper quadrantectomy and sentinel lymph node biopsy. The seroma developed on postoperative day 7 was managed by office-based aspiration and had completely resolved by day 15.

**Figure 2:**
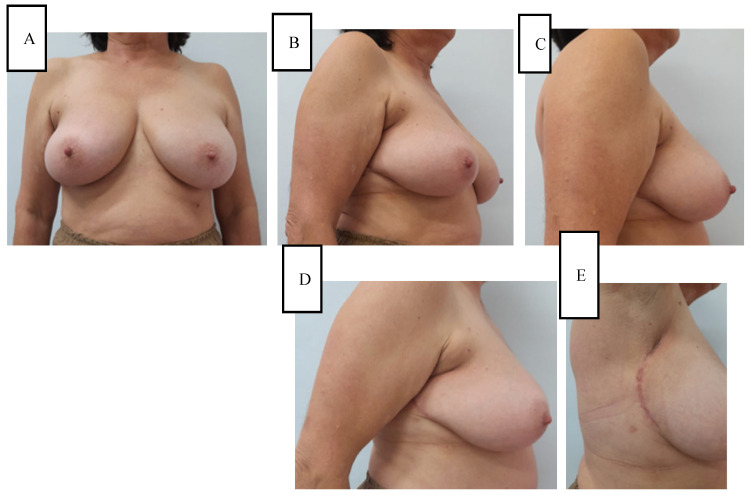
(**A**–**C**) Six months postoperative photos of the patient in Figure 1. Note the perfect breast symmetry and the natural appearance of the breast. (**D**,**E**) Detail of the post-surgical scar. Note how it is hidden by the arm.

**Figure 3:**
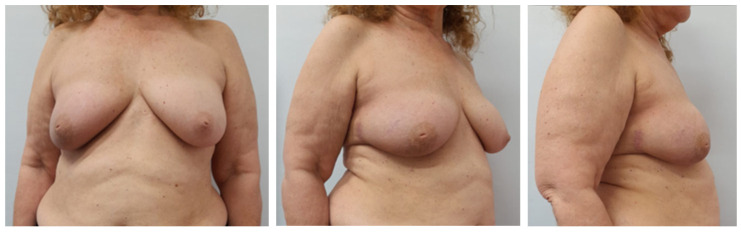
Six months postoperative photos in a patient who underwent right superouterine quadrantectomy and reconstruction with LICAP flap.

Cosmetic evaluation at six months, performed independently by two senior breast surgeons using the Harvard four-point scale, rated the outcome as excellent in 7 of 9 (78%) breasts and good in 2 of 9 (22%), with an inter-rater agreement of κ = 0.81. Mild donor-site hypoesthesia was noted in 2 of 9 (22%) women and resolved spontaneously by the 6-month follow-up. No other donor-site morbidity (such as dog-ear, contour deformity, or hypertrophic scarring) was observed. To illustrate these outcomes, representative six-month postoperative results are shown in Figure 2 and Figure 3.

Patient-reported satisfaction as measured by the BREAST-Q^®^ Version 2.0 Breast-Conserving Therapy Module reached a mean of 79 ± 12 on the 0–100 scale. A domain-level breakdown of BREAST-Q® scores is reported in Table 2. Subdomain analysis showed high satisfaction across most domains, particularly for postoperative breast satisfaction (82 ± 11), psychosocial well-being (71 ± 8), and satisfaction with the breast unit team (80 ± 13). Physical well-being of the chest scored 20 ± 6, where a lower score reflects a low occurrence of postoperative pain and discomfort. Scores for sexual well-being and cancer worry were 68 ± 6 and 36 ± 4, respectively. These findings suggest that while reconstructive outcomes were consistently positive in aesthetic and functional terms, residual cancer-related concerns persisted for some patients.

## 4. Discussion

The results of our initial experience demonstrate that the lateral intercostal artery perforator flap is a safe and effective option for Level II oncoplastic breast reconstruction, particularly for lateral and central–lateral defects. In our cohort of nine patients, we observed a single minor flap-related complication (11%), represented by a postoperative seroma that resolved with aspiration and did not require re-operation or delay in adjuvant therapy. Importantly, no cases of necrosis, flap loss, infection, or wound dehiscence occurred, and aesthetic and patient-reported outcomes at six months were excellent. These findings are consistent with existing literature highlighting the LICAP flap’s favourable complication profile, reproducibility, and high patient satisfaction [7,8]. Beyond confirming the safety of the technique, our study adds value by combining surgeon-based evaluation with patient-reported outcomes using the BREAST-Q. This dual assessment provides a more comprehensive picture of functional and aesthetic results in the early postoperative period and helps to contextualize the role of LICAP flaps in everyday practice. Patient-reported outcomes are increasingly recognized as essential endpoints in oncoplastic research, since they provide unique insight into quality of life domains such as body image and psychosocial recovery, which cannot be fully captured by surgeon-based evaluations. From this standpoint, LICAP reconstruction illustrates how oncoplastic surgery can incorporate precision principles, offering tailored solutions that align oncologic safety with functional and aesthetic priorities unique to each patient.

Orabi et al. [7] reported an 88.5% patient satisfaction rate and minimal early complications in a prospective cohort of 26 patients undergoing lateral chest wall perforator flap reconstruction. Similarly, Hashem et al. [9] found no significant differences in complication rates between LICAP and TDAP flaps. Still, they noted the LICAP flap was technically more straightforward, with more consistent perforators and reduced need for intraoperative repositioning. Meybodi et al. [6] described a modified LICAP approach in 22 patients and reported a single case of wound infection requiring surgical washout (5%), further reinforcing the low morbidity associated with the technique. Our shorter operative time (mean 128 min) and blood loss (85 mL) also compare favourably with published averages for volume replacement flaps, reflecting the LICAP flap’s surgical efficiency.

The LICAP flap offers several distinct advantages over alternative techniques. Its reliable perforator anatomy, typically from the 5th to 8th intercostal spaces, enables flap harvest without sacrificing underlying muscle, thereby preserving shoulder function and reducing donor-site morbidity [5,6]. The supine position during harvest facilitates operative workflow and eliminates the need for intraoperative repositioning, which contrasts with other regional or free flap techniques. Furthermore, the donor site is usually concealed along the bra line and can be closed primarily without tension, leading to high aesthetic satisfaction and minimal contour irregularities. In our study, 100% of patients achieved a Harvard score of ‘good’ or ‘excellent’ at six months, and the mean BREAST-Q satisfaction score of 79 corroborates the favourable patient experience reported by Carmichael et al. [4]. Nonetheless, the flap is not without limitations; in very slim patients, the available donor volume may occasionally be limited, and in cases requiring more medial reach, additional dissection or hybrid techniques can be necessary. Furthermore, the learning curve for reliable perforator identification and preservation requires familiarity with perforator-based techniques.

When compared with other local perforator flaps, particularly the anterior intercostal artery perforator (AICAP) flap, the LICAP flap demonstrates superior versatility, technical reliability, and aesthetic outcomes in the context of lateral and central–lateral breast defects. The AICAP flap, which is based on perforators emerging near the parasternal region, is generally limited in arc of rotation and provides shorter pedicle length, making it more suitable for lower inner quadrant reconstructions [8]. In contrast, the LICAP flap’s posterior origin allows for a longer pedicle and a broader arc of rotation, enabling more effective coverage of lateral, central, and even select medial defects through careful tunnelling or transposition techniques [11]. Additionally, AICAP harvest requires careful consideration to avoid injuring the internal mammary vasculature, particularly in patients who may later require free flap reconstruction. In contrast, the LICAP flap spares the thoracodorsal and internal mammary systems, preserving future reconstructive options. Compared to more centrally based flaps like the LTAP (lateral thoracic artery perforator) flap, the LICAP also provides a more predictable anatomical course and typically allows for supine-only harvest, streamlining intraoperative workflow. These characteristics position the LICAP flap as a workhorse for volume replacement in breast-conserving surgery, especially when technical reproducibility, patient comfort, and future reconstructive planning are considered. In our series, the technical aspects of the procedure also contributed to favourable outcomes. The supine-only harvest and reliable perforator anatomy allowed for efficient flap elevation without the need for patient repositioning, which reduced operative complexity. Recovery was generally fast, with patients discharged after a median of one night and drains removed within a few days. Patient satisfaction and aesthetic results were high, in line with previously published studies reporting comparable outcomes with LICAP and related perforator flaps [4,7,9]. These findings further support the functional safety and cosmetic reliability of the technique in appropriately selected patients.

The primary strengths of our study lie in the consistency of surgical technique, single-centre control, and standardised follow-up using objective (Harvard scale) and validated patient-reported outcome measures (BREAST-Q). All operations were performed by the same experienced oncoplastic team, reducing procedural variability. Furthermore, our inclusion criteria were clearly defined, focusing on well-selected patients with lateral or central-lateral defects amenable to LICAP reconstruction. However, the study is limited by its small sample size and retrospective design, which preclude statistical hypothesis testing or generalizability to broader populations. While adequate to assess early surgical and aesthetic outcomes, our follow-up duration does not permit conclusions regarding long-term oncologic safety or durability of the reconstruction. Although no local or distant recurrences were observed during a median follow-up of 10 months, longer surveillance is required to confirm the oncologic durability of the LICAP flap in the setting of breast-conserving surgery. Like other LICAP studies to date [8,12], ours remains a single-institution experience that should be interpreted within the context of these limitations. Nevertheless, our findings support the integration of LICAP flaps into routine surgical planning for appropriately selected patients, particularly in centres aiming to expand their armamentarium of muscle-sparing oncoplastic techniques. While the number of patients in this series is limited, the cohort was consecutive and homogeneous, with outcomes assessed through both objective scales and validated patient-reported measures. This dual evaluation not only strengthens the reliability of our outcomes but also reflects a personalized approach, as it integrates both clinical judgment and patient experience in defining success. This provides consistent data that contribute to understanding the role of LICAP flaps in oncoplastic breast surgery. In addition, recent reports have further contributed to expanding the evidence base of LICAP and related chest wall perforator flaps, highlighting their reproducibility, favourable complication profile, and high patient satisfaction [13,14].

Future research should, therefore, focus on prospective multicentre cohorts that use validated aesthetic panels and BREAST-Q^®^ modules at serial time points to generate granular longitudinal data. Randomised controlled trials comparing LICAP with thoracodorsal artery perforator or extended glandular rotation flaps would clarify relative cost-effectiveness, functional morbidity, and patient preference. Imaging-based perfusion studies and computational modelling could deepen our understanding of flap behaviour under hypofractionated and partial-breast irradiation schedules. Finally, biomechanical analyses of shoulder function after various donor-site options would refine patient selection algorithms.

## 5. Conclusions

The LICAP flap is a safe, efficient, and cosmetically superior volume-replacement technique for lateral-quadrant defects in breast-conserving oncoplastic surgery. Its minimal donor-site morbidity, rapid harvest, and excellent early aesthetic outcomes support its role as a first-line option in appropriately selected patients. Importantly, the LICAP flap allows a personalized reconstructive strategy tailored to individual anatomy and defect characteristics [15]. The combined use of surgeon-based aesthetic scales and validated patient-reported outcomes in our series further supports the reliability of these findings and highlights the importance of integrating both perspectives in evaluating surgical success. In clinical practice, the LICAP flap can expand the reconstructive repertoire of breast units by offering a reproducible, muscle-sparing solution that preserves future reconstructive options. Considering the retrospective design and the small sample size, this work can be regarded as a pilot study. Larger prospective studies with extended follow-up are needed to validate and refine its indications. In this sense, LICAP flaps represent a concrete example of precision oncoplastic surgery, where reconstructive planning is individualized to optimize outcomes for each patient. As evidence accumulates, they are poised to become a reference technique within the spectrum of Level II oncoplastic procedures, bridging oncologic safety with cosmetic reliability and patient-centred care.

## Figures and Tables

**Table 1:** Baseline, operative, and outcome data (n = 9).

Age, Years—Median (Range)	55 (37–68)
BMI, kg m^2^—median (IQR)	24.3 (22.1–27.6)
Current smokers, n (%)	3 (33%)
Diabetes mellitus, n (%)	2 (22%)
Tumour size, mm—median (IQR)	17 (12–22)
Tumour quadrant, n	2 upper-outer; 7 central–lateral
Pathological stage, n	6 pT1c; 3 pT2
Sentinel nodes retrieved—median (range)	2 (1–3)
Positive sentinel nodes, n	0
Operative time, min—mean ± SD	128 ± 15
Estimated blood loss, ml—mean ± SD	85 ± 20
Hospital stay, days—median (IQR)	1 (1–2)
Flap complications (0–90 d)	1 (11%)
Re-operations	0
Harvard scale ≥ good, n (%)	9 (100%)
BREAST-Q^®^ satisfaction—mean ± SD	79 ± 12
Donor-site hypo-aesthesia, n (%)	2 (22%)
Median follow-up, months (range)	10 (6–14)
Local/distant recurrences	0

**Table 2:** BREAST-Q^®^ Version 2.0 Breast-Conserving Therapy Module.

N.	Breast Q Subdomain	Mean ± SD
1	Satisfaction with Information—Breast Surgeon	75 ± 9
2	Satisfaction with Medical Team	90 ± 9
3	Satisfaction with Office Staff	85 ± 13
4	Satisfaction with Surgeon	87 ± 10
5	Satisfaction with Information—Radiation Oncologist	68 ± 6
6	Psychosocial Well-Being	71 ± 8
7	Sexual Well-Being	68 ± 6
8	Cancer Worry	36 ± 4
9	Satisfaction with Breasts (Preoperative)	66 ± 16
10	Satisfaction with Breasts (Postoperative)	82 ± 11
11	Physical Well-Being: Chest (Preoperative)	7 ± 5
12	Physical Well-Being: Chest (Postoperative)	20 ± 6
* Total satisfaction 79 ± 12		

* Note: this value is calculated excluding subdomains 8, 9, 11, and 12.

## Data Availability

The data presented in this study are available on reasonable request from the corresponding author. The data are not publicly available due to privacy and ethical restrictions.

## Supplementary Materials

The following supporting information can be downloaded at: https://www.mdpi.com/article/10.3390/jpm15100491/s1, Video S1: Surgical technique.

## Abbreviations

The following abbreviations are used in this manuscript:

BCS	Breast-conserving surgery
OBS	Oncoplastic breast surgery
Licap	Lateral intercostal artery perforator
TDAP	Thoracodorsal artery perforator
AICAP	Anterior intercostal artery perforator
LTAP	Lateral thoracic artery perforator
BMI	Body mass index
NACT	Neoadjuvant chemotherapy
SD	Standard deviation
IQR	Interquartile range
BREAST-Q	Breast-Q^®^ Patient-Reported Outcome Measure
